# An Update: Enzymatic Synthesis for Industrial Applications

**DOI:** 10.1002/anie.202505976

**Published:** 2025-05-16

**Authors:** Thomas Bayer, Shuke Wu, Radka Snajdrova, Kai Baldenius, Uwe T. Bornscheuer

**Affiliations:** ^1^ Institute of Biochemistry, Dept. of Biotechnology & Enzyme Catalysis Greifswald University Felix‐Hausdorff‐Str. 4 17487 Greifswald Germany; ^2^ National Key Laboratory of Agricultural Microbiology, College of Life Science and Technology Huazhong Agricultural University 1 Shizishan Street Wuhan 430070 P.R. China; ^3^ Novartis Institutes for BioMedical Research Global Discovery Chemistry Basel 4056 Switzerland; ^4^ Baldenius Biotech Consulting Hafenstr. 31 68159 Mannheim Germany

**Keywords:** Biocatalysis, Enzyme catalysis, Industrial catalysis, Organic synthesis, Stereoselectivity

## Abstract

Supported by rapid technological advancements, biocatalytic applications have matured into sustainable, scalable, and cost‐competitive alternatives to established chemical catalysis. This review presents the most recent examples of enzyme‐based solutions for the manufacturing of molecules with extended carbon–carbon frameworks and multiple stereogenic centers at commercial scale, including peptide building blocks, (rare) sugars, synthetic (oligo)nucleotides, and terpenoids, such as (–)‐Ambrox^®^. Novel enzyme classes are highlighted along with their potential applications—the synthesis of DNA/RNA, the depolymerization of synthetic plastics, or fully enzymatic protection/deprotection schemes—pointing toward the diversification and broader industrial utilization of biocatalysis‐based processes.

## Graphical abstract

Biocatalysis has become a sustainable and cost‐competitive alternative to established chemical synthesis, enabling the enzyme‐based production of not only commodity chemicals but (non‐natural) amino acids, (rare) sugars, as well as synthetic nucleotides. These building blocks give access to highly complex molecules with versatile industrial and therapeutic applications.

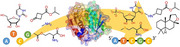

## 1 Introduction

Since our review article from 2021^[^ ^1^ ^]^ which critically assessed the utilization of enzymatic synthesis for industrial applications and was well received by the scientific community, there has been significant progress in the area of biocatalysis.

In this update, we thus summarize recent examples on functional group transformations yielding highly desired (chiral) products, including amines, alcohols, carboxylic acid derivatives, as well as complex molecules like carbohydrates and nucleic acids. Importantly, we highlight emerging classes of enzymes for novel applications such as the biocatalytic (de)protection of functional groups and the depolymerization of (synthetic) plastics—of which some have already crossed the barrier to larger‐scale applications. We complement excellent recent reviews on biocatalysis, for example, by Bell et al.,^[^ ^2^ ^]^ Lovelock et al.,^[^ ^3^ ^]^ Hanefeld et al.,^[^ ^4^ ^]^ O'Connell et al.,^[^ ^5^ ^]^ France et al.,^[^ ^6^ ^]^ as well as a book chapter by Wohlgemuth.^[^ ^7^ ^]^ Phelan et al. covered enzyme‐based, scalable processes using nitrilases, Baeyer–Villiger monooxygenases, and imine reductases, besides other biocatalysts.^[^ ^8^ ^]^ Similarly, Grandi et al. highlighted enzymatic oxy‐ and amino‐functionalization using cascade reactions.^[^ ^9^ ^]^ The strengths and limitations of enzymatic late‐stage modifications have been summarized too.^[^ ^10^ ^]^ In a review just published, the importance of both chemical and biocatalytic process development in the pharmaceutical industry in Europe was summarized.^[^ ^11^ ^]^ Furthermore, technological advancements for enzyme discovery and design, but also for both tuning and scaling biocatalytic reactions have been the focus of a recent special issue^[^ ^12^ ^]^ and a hot topic at the “*Faraday discussions”* held in London in 2024.^[^ ^13^, ^14^, ^15^, ^16^, ^17^ ^]^ Additionally, a collection of research works focused on recent developments within the interdisciplinary field of chemoenzymatic synthesis.^[^ ^18^ ^]^ Readers, particularly interested in key performance indicators (KPIs) for industrial applications as well as reasons why certain biocatalytic routes may have not been commercialized on scale, are kindly referred to our review from 2021 as our statements made there are still valid. Along this line, enzymes that can catalyze new‐to‐nature chemistry, for example, by the incorporation of non‐canonical amino acids^[^ ^19^ ^]^ or the use of artificial metalloenzymes,^[^ ^20^ ^]^ as well as synthetic CO_2_ fixation concepts^[^ ^21^ ^]^ are out of the scope of this article as industrial implementation might be challenging in the near future. Nonetheless, these breakthroughs not only explore and expand the diversity of biocatalytic processes; they demonstrate the importance of sophisticated tools for the computational design of proteins and enzymes,^[^ ^22^ ^]^ as well as structure prediction methods developed by Baker,^[^ ^23^ ^]^ Hassabis and Jumper,^[^ ^24^, ^25^ ^]^ who won the Nobel Prize in Chemistry 2024.^[^ ^26^ ^]^ Together, these tools have already been used to cater industrial biocatalysts and they will certainly aid to propel novel enzymes into application in the future, as discussed in the following.

## 2 Recent Examples of Improved Biocatalysts

### 2.1 Synthesis of Amines

**Scheme 1:**
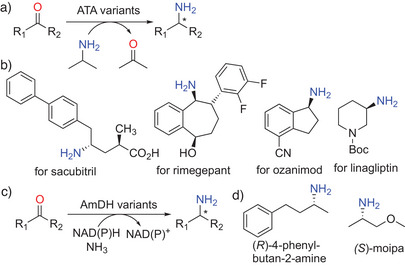
ATA‐ and AmDH‐catalyzed synthesis of optically pure primary amines. a) Transformation of ketones into the corresponding chiral amines by ATA (variants) at the expense of isopropylamine as the amine donor. b) Selected amine intermediates toward the manufacturing of pharmaceuticals through ATA‐driven catalysis. c) AmDH (variants) use cheap ammonia for asymmetric reductive aminations. d) Products of upscaled AmDH‐catalyzed reactions.

Chiral amines remain pivotal intermediates and target products in the synthesis of pharmaceuticals and bioactive compounds (Scheme 1). One hallmark of implementing enzyme catalysis in an industrial process was the use of an engineered amine transaminase (ATA) in the asymmetric synthesis of Sitagliptin by Codexis in 2010,^[^ ^27^ ^]^ besides other showcase examples.^[^ ^1^ ^]^ Since then, ATA‐mediated processes for chiral amine production, including cascade reactions, have further matured.^[^ ^28^, ^29^, ^30^ ^]^ Importantly, fundamental thermodynamic and kinetic aspects of ATA‐catalyzed reactions have recently been reported.^[^ ^31^ ^]^ A prominent trend in ATA utilization to convert challenging substrates like—but not limited to—bulky heterocycles has been the data‐driven prediction and engineering of activities of ATA variants (Scheme 1a).^[^ ^32^, ^33^ ^]^ Complementarily, the development of ultrahigh‐throughput assays (e.g., based on growth selection)^[^ ^34^ ^]^ has enabled the fast identification of desired hits in directed evolution campaigns. The successful identification of enantio‐complementary ATAs and their engineering toward enhanced activities yielded potent biocatalysts for the manufacturing of chiral key amine intermediates for a range of compounds such as sacubitril (75 g l ^−1^ substrate concentration, 90% conversion, perfect enantioselectivity),^[^ ^35^ ^]^ rimegepant (kilogram scale, 99.5%de, >99% purity),^[^ ^36^ ^]^ linagliptin (100 g l ^−1^ substrate concentration, 74% yield, space‐time yield (STY): 10 g l ^−1^ *h),^[^ ^37^ ^]^ and ozanimod (69% yield, >99%ee; Scheme 1b).^[^ ^38^ ^]^

From a practical perspective, the ATA‐catalyzed synthesis of primary amines still faces two major challenges: the relatively high cost of (organic) amine donors and the requirement for pyridoxal‐5′‐phosphate (PLP)‐dependent ATAs to accommodate two distinct substrates (donor and acceptor). The employment of amine dehydrogenases (AmDHs), pioneered by the Bommarius group in 2012, offers a potentially more economical alternative through asymmetric reductive amination using ammonia as the nitrogen source (Scheme 1c).^[^ ^39^, ^40^, ^41^ ^]^

Over the past five years, significant efforts have been devoted to the discovery and tailoring of novel AmDHs. Protein engineering campaigns greatly enhanced the substrate scope of AmDHs toward alkyl (hetero)aryl ketones, fused/linked‐ring aryl ketones, and *N*‐heterocyclic primary amines.^[^ ^42^, ^43^, ^44^ ^]^ Notably, the recently engineered thermostable CalAmDH‐M3_1_ has demonstrated remarkable potential in the kilogram‐scale synthesis of (*R*)‐4‐phenylbutan‐2‐amine, achieving >99.9%ee and 85% isolated yield at a substrate concentration of 120 g l ^−1^ with oleic acid serving as both activator and stabilizer for the AmDH.^[^ ^45^ ^]^ These developments clearly indicate the potential of AmDH‐mediated processes for the efficient synthesis of primary amines. One remaining challenge is that most AmDHs originate from natural leucine/phenylalanine dehydrogenases and current processes predominantly yield the (*R*)‐enantiomers of primary amines (Scheme 1d). The discovery of a number of natural AmDHs by Vergne‐Vaxelaire et al. promises to access a broader range of products at higher efficiency^[^ ^46^ ^]^—even though first small scale demonstrations on the benchmark molecule (*S*)‐moipa had a low STY (0.3 g l ^−1^ *h at 42 g pure AmDH/kg product; 88% conversion)^[^ ^47^ ^]^ compared to Celgene's ATA‐ or BASF's lipase‐based approaches.^[^ ^1^ ^]^

**Scheme 2:**
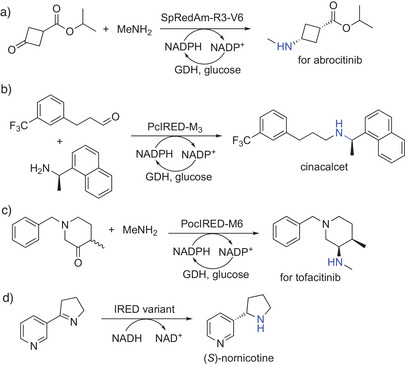
RedAm‐ and IRED‐catalyzed synthesis of optically pure secondary amines. Commercialized processes for the manufacturing of a) an amine intermediate toward abrocitinib, b) cinacalcet from 3‐[3‐(trifluoromethyl)phenyl]propanal and (*R*)‐1‐(1‐naphthyl)ethanamine, c) a tofacitinib precursor, and d) (*S*)‐nornicotine.

Equally important to the synthesis of (chiral) primary amines, is the manufacturing of secondary and tertiary amines through the reductive amination of various carbonyl compounds.^[^ ^48^ ^]^ In this regard, imine reductases (IREDs) and reductive aminases (RedAms) have gained attention.^[^ ^48^, ^49^ ^]^ Despite their mechanistical similarity, RedAms and IREDs can be distinguished through their primary sequence; RedAms are a subset of IREDs, capable of binding both carbonyl and amine substrates to facilitate the formation of the imine intermediate in their active sites prior to the enantioselective imine hydrogenation.^[^ ^49^ ^]^ Following the discovery of IREDs and RedAms through the screening of microbial strains and metagenomic libraries,^[^ ^50^ ^]^ particularly RedAms have been tailored to facilitate transformations at kilogram scales.^[^ ^48^ ^]^ One of the outstanding industrial examples is the synthesis of *cis*‐isopropyl 3‐(methylamino)cyclobutane‐1‐carboxylate (>99:1 *cis*:*trans*), a key intermediate for abrocitinib, in commercial manufacture at multi‐tons with a STY of 2.5 g L^−1^ h^−1^ by using an engineered RedAm developed by Pfizer (Scheme 2a).^[^ ^51^ ^]^ Very recent examples featured the synthesis of the anti‐hyperparathyroidism drug cinacalcet with a STY of 3.2 g l ^−1^ *h, 85% yield, and excellent optical purity (>99.0%ee), using an engineered IRED and a high substrate load (>0.5 m; Scheme 2b).^[^ ^52^ ^]^ An IRED has been employed to construct contiguous stereocenters, which was efficiently demonstrated in the recent synthesis of a key intermediate of tofacitinib at a substrate loading of 110 g l ^−1^ (74% yield, *>*99.9%ee, and 98:2 dr; Scheme 2c).^[^ ^53^ ^]^ For the synthesis of (*S*)‐nicotine, the company Zanoprima, together with Enzymicals AG, developed a process running on the multi‐ton scale using an IRED, which converts myosmine into the desired (*S*)‐nornicotine with excellent enantioselectivity (>99%ee at full conversion; Scheme 2d); a subsequent chemical step then yields the (*S*)‐nicotine.^[^ ^54^ ^]^ For comparison, the current chemical process suffers from low yields (∼25%) and involves the hydrogenation of myosmine with a metal catalyst, followed by the selective crystallization of the biologically active enantiomer using dibenzoyl tartrate (2 kg is needed to resolve 1 kg of (*S*)‐nicotine).^[^ ^55^ ^]^

Another example is the use of an engineered RedAm for the synthesis of a key intermediate of a CDK 2/4/6 inhibitor, where the chiral amine product was obtained in 35% isolated yield and 98.4%ee.^[^ ^56^ ^]^ A very recent example described the synthesis of an intermediate of avacopan using an IRED (and an ADH for cofactor recycling). This set‐up enabled the control of two vicinal stereogenic centers in an *N*‐heterocyclic system and resulted in a kilogram‐scale synthesis with a total yield of 58%, 99.5%ee, and a STY of 1.5 g l ^−1^*h.^[^ ^57^ ^]^

As emerging classes of enzymes, AmDHs, IREDs, and RedAms have yet to be more broadly implemented in up‐scaled manufacturing processes. As oxidoreductases, they require easy‐to‐regenerate nicotinamide cofactors (Scheme 2),^[^ ^58^ ^]^ which in contrast to transaminases, has the advantage of avoiding reaction equilibrium and as such no large excess of ammonia is needed when primary amines are produced, leading also to an easier work up procedure. Alkyl, aryl, and cyclic amines can be used to access secondary and tertiary amines.^[^ ^48^ ^]^ Recent advances also included engineered AmDHs in cascade‐type reactions to produce non‐canonical amino acids, an important compound class described below.^[^ ^59^ ^]^ This versatility will certainly make them a valuable addition to the toolbox of industrial biocatalysts, besides the well‐established ATAs, to access complex chiral molecules with one or even multiple stereocenters.

### 2.2 Synthesis of Carboxylic Acids and Derivatives

Optically pure carboxylic acids, carboxylic amides, amino acids, as well as structurally related derivatives are widely used across many different industries.^[^ ^1^, ^60^ ^]^

**Scheme 3:**
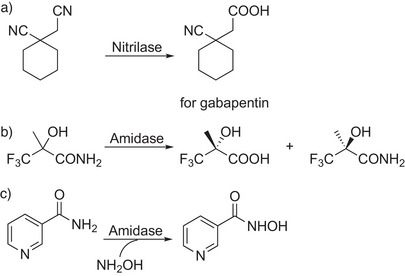
Amidase‐catalyzed reactions. Manufacturing of a) a gabapentin precursor, b) (*R*)‐3,3,3‐trifluoro‐2‐hydroxy‐2‐methylpropionic acid, and c) nicotinyl hydroxamic acid with high STYs. Water molecules are not shown.

In our previous review, we focused on the hydrolysis of esters and selected amides by well‐established biocatalysts like esterases and lipases for the production of (chiral) carboxylic acids. Industrial processes, employing nitrilase‐based or nitrile hydratase/amidase systems, have yielded valuable compounds like nicotinic acid^[^ ^61^ ^]^ or acrylamide on multi‐ton scales.^[^ ^62^ ^]^ Currently, the remarkable selectivity of nitrilases is applied beyond racemate resolutions. Xue et al. hydrolyzed 1‐cyanocyclohexylacetonitrile with a nitrilase from *Acidovorax facilis* to the mononitrile precursor for gabapentin (19 g l ^−1^ *h with 89 g (cell dry weight) *E. coli* per kg mononitrile acid, Scheme 3a).^[^ ^63^ ^]^ Similarly, BASF uses a nitrilase from *Comamonas testosteroni* to hydrolyze terephthalodinitrile to 4‐cyanobenzoic acid, a building block for fungicides.^[^ ^64^ ^]^ In both cases, very high selectivities (up to 99.8%) for mono‐hydrolysis are observed at (almost) full conversion. Only 2 g (cell dry weight) of *E. coli* is required per kg of 4‐cyanobenzoic acid to achieve a 9 g L^−1^*h STY.

Very recently, various amidase enzymes have regained attention for the biodegradation of inert plastic materials, including polyamides (PAs) and polyurethanes (PUs) as discussed below in Section 2.9. Previously, amidases have been successfully used for (dynamic) kinetic resolutions, yielding enantiopure carboxylic acids and amides,^[^ ^65^, ^66^ ^]^ as well as a variety of optically pure d‐ and l‐amino acids.^[^ ^60^, ^67^, ^68^ ^]^ The kinetic resolution of (*R*)‐3,3,3‐trifluoro‐2‐hydroxy‐2‐methylpropionic acid, which is an important pharmaceutical intermediate for the synthesis of pyruvate dehydrogenase kinase inhibitors and pain killers,^[^ ^69^ ^]^ could be drastically improved by the use of an amidase, with a substrate load of 200 g l ^−1^ and a very short reaction time of only 10 min (Scheme 3b).^[^ ^70^ ^]^ Furthermore, 2‐chloronicotinic acid^[^ ^71^ ^]^ and the gabapentin precursor (1‐cyanocyclohexanoic acid from 1‐cyanocyclohexaneacetamide)^[^ ^1^, ^60^ ^]^ were synthesized by (engineered) amidase signature (AS) family enzymes with remarkable STYs (24 g l ^−1^ *h^[^ ^71^ ^]^ and ∼240 g l ^−1^ *h,^[^ ^72^ ^]^ respectively).

Complementary to the hydrolysis of target substrates described in our previous review and above, the promiscuous acyltransferase activity of esterases and amidases, as well as the activity of different adenosine triphosphate (ATP)‐dependent enzymes can be exploited to form ester and amide bonds as highlighted recently.^[^ ^60^, ^73^, ^74^, ^75^ ^]^ Regarding the manufacturing of flavor esters, the acyltransferase *Ms*AcT was used for the acylation of primary alcohols (e.g., isoamyl, *n*‐hexyl, geranyl, benzyl, 2‐phenylethyl, and cinnamyl alcohol) in aqueous solutions with substrate loads of up to 0.5 m and reasonable STYs.^[^ ^76^ ^]^ Different acylation reactions, yielding esters but also amides like melatonin and other value‐added tryptamine derivatives, could be intensified by using flow systems with immobilized *Ms*AcT^[^ ^73^, ^77^, ^78^ ^]^ but will need further improvements to ultimately meet industrial metrics.^[^ ^1^, ^79^ ^]^ The same accounts for a novel biocatalytic cascade reaction reported by the group of Micklefield, transforming readily available organic nitriles by a combination of nitrile‐hydrolyzing enzymes, amide bond synthetases, and photoredox catalysis.^[^ ^80^ ^]^ Complementary, the acyl transfer activity of an amidase from *Pseudomonas putida* BR‐1 efficiently converted 0.2 m nicotinamide and 0.25 m hydroxylamine‐HCl to nicotinyl hydroxamic acid (STY: 32 g l ^−1^ *h; Scheme 3c).^[^ ^81^ ^]^ Generally, hydroxamic acid derivatives have versatile applications in medicine, agriculture, and as food additives, for example.^[^ ^1^, ^60^ ^]^

**Scheme 4:**
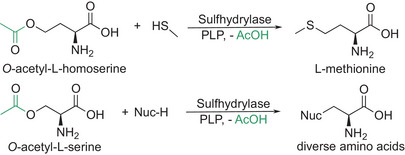
Synthesis of l‐amino acids using sulfhydrylases. The PLP‐dependent activation of amino acid derivatives can yield natural amino acids like l‐Met (top) or various non‐natural amino acids (bottom).

Due to the industrial importance of amino acids as building blocks for pharmaceuticals and (therapeutic) peptides,^[^ ^82^, ^83^ ^]^ advances beyond the readily established conversion of α‐keto acids into α‐amino acids will be discussed next.^[^ ^84^, ^85^ ^]^Amongst the natural amino acids, methionine is the third largest amino acid with an annual production of at least 1.5 million tons, predominantly for animal nutrition. In contrast to other high‐volume amino acids like l‐Glu and l‐Lys, it is still mostly produced by conventional chemistry (Strecker synthesis) and delivered as d,l‐racemate. Fermentative production of l‐Met has traditionally been hampered by the challenge to introduce sulfur. The company CJ CheilJedang, in partnership with Arkema (providing sulfur chemistry expertise), has overcome this bottleneck by fermenting the methionine precursor *O*‐acetyl‐l‐homoserine to high titers and substituting the terminal acetate group with methyl mercaptan, using a sulfhydrylase‐catalyzed step in vitro (Scheme 4).^[^ ^86^, ^87^ ^]^ This process has been industrialized on the 80 000 t a^−1^ scale.^[^ ^88^, ^89^ ^]^ Sulfhydrylases have attained relatively little attention in the preparative biocatalysis community despite their remarkable potential. Sulfhydrylases can guide the activation of the amino acid by PLP to enable substitution on either the γ‐position (as for methionine) or on the β‐position (as for cysteine)^[^ ^90^ ^]^ and can yield several non‐natural amino acids.^[^ ^91^ ^]^

Noteworthily, the mechanism regarding the γ‐substitution of *O*‐acyl‐homoserine has recently been reviewed^[^ ^91^, ^92^ ^]^ and the mechanism of the β‐substitution in *O*‐acyl‐serine derivatives is also well‐known.^[^ ^93^ ^]^

**Scheme 5:**
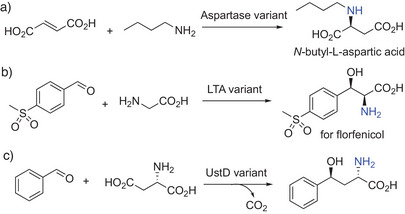
Enzymatic synthesis of non‐natural l‐amino acids. Protein engineering facilitated and greatly improved the synthesis of a) *N‐*butyl‐l‐aspartic acid, b) an intermediate toward florfenicol, or c) (4*S*)‐4‐Phenyl‐l‐homoserine.

Further significant advancements in the past 5 years include the synthesis of β‐branched non‐canonical amino acids using β‐selective amino acid transaminases^[^ ^94^, ^95^ ^]^ or engineered ammonia lyases.^[^ ^96^ ^]^ Aspartase has been engineered to eventually catalyze the simple addition of ammonia to acrylic acid for the formation of β‐alanine, a multi thousand tons intermediate for vitamin B5 production.^[^ ^97^, ^98^, ^99^, ^100^ ^]^ Although the metrics of this addition have not yet reached industrial standards, the success of aspartase leaves hope for this cost efficient synthetic route to β‐alanine. Additionally, the synthesis of various *N*‐substituted β‐amino acids from unsaturated carboxylic acids and amines (via hydroamination) has been achieved, using computationally redesigned aspartase variants.^[^ ^101^ ^]^ Notably, *N*‐butyl‐l‐aspartic acid, a precursor to a neotame (sweetener) analogue, was synthesized with high optical purity and yield on a kilogram scale (Scheme 5a). Another important advancement in chiral amino acid synthesis involves carbon–carbon bond formation. For instance, l‐threonine aldolase (LTA) has been engineered to enhance or invert its diastereoselectivity, enabling the synthesis of chiral β‐hydroxy‐α‐amino acids with high diastereoselectivity of 99.4%_syn_ and an inverted de value of 97.2%_anti_.^[^ ^102^ ^]^

This LTA‐mediated process has been successfully applied on a commercial scale to replace a traditional chemical resolution process to manufacture l‐*syn*‐3‐[4‐(methylsulfonyl)phenylserine], a key precursor for florfenicol (Scheme 5b). Another enzyme, UstD, catalyzes decarboxylative aldol reactions for the synthesis of γ‐hydroxy‐α‐amino acids.^[^ ^103^ ^]^ Through directed evolution and semi‐rational engineering, UstD was optimized for improved activity toward aromatic substrates, enabling access to amino acids with γ‐tertiary alcohols and cyclic imino acids (Scheme 5c).^[^ ^104^ ^]^

**Scheme 6:**
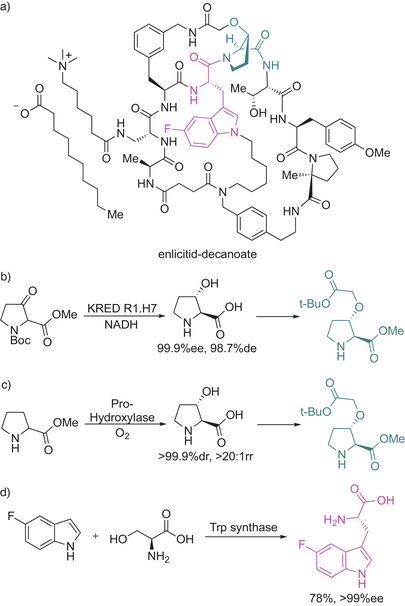
Enlicitid synthesis. a) Structure of enlicitid decanoate with the two building blocks highlighted. b) Initial route to make the 3‐*trans* hydroxyproline using a ketoreductase (KRED) route. c) Improved route to make the 3‐*trans* hydroxy proline using an α−KG‐dependent Pro‐hydroxylase, and d) tryptophan synthase‐catalyzed route to afford the fluorinated Trp analog.^[^ ^105^ ^]^

A great manifest for the enzymatic synthesis of non‐canonical amino acids (ncAAs) is the recent example about the second‐generation process toward the macrocyclic peptide enlicitid (Scheme 6), published by Merck & Co., Inc. recently.^[^ ^105^ ^]^ Its structure contains several ncAAs and other nonpeptidic motifs, which presents significant synthetic challenges. 3‐*trans* hydroxyproline (shown in green, Scheme 6) was prepared in the original route from simple commercial building blocks (methyl glycinate and ethyl acrylate) and chiral separation to isolate the desired enantiomer. Through improvements using a ketoreductase (KRED)‐catalyzed dynamic kinetic diastereoselective reduction of the corresponding 3‐keto proline, the team pursued an even more productive way to prepare 3‐*trans* hydroxyproline via direct installation of the desired hydroxy group at the 3‐position of the l‐proline ring by using a α‐ketoglutarate‐dependent proline hydroxylase.

This achievement alone was estimated to improve the metrics of the process to an extent that only 100 kg of l‐proline was necessary to make 100 kg of enlicitid instead of procuring 170 MT of methyl glycinate and ethyl acrylate to synthesize the same amount of enlicitid. Thanks to an additional enzymatic step (using a tryptophan synthase giving access to the modified tryptophan; shown in pink, Scheme 6) and an enormous process development effort, this ensured a convergent, efficient, and robust manufacturing process enabling large‐scale production of enlicitid with a final synthetic route of 43 steps.^[^ ^105^ ^]^

Beyond natural enzymatic reactions, innovative approaches have emerged, such as coupling PLP‐dependent enzymes with photoredox catalysts to achieve oxidative cross‐coupling reactions.^[^ ^106^, ^107^ ^]^ Such strategies, albeit pending industrial utilization, have expanded the access to a diverse range of non‐natural amino acids, demonstrating the versatility and potential of enzyme‐mediated synthesis in modern chemical biology and pharmaceutical production.

### 2.3 Synthesis of (Chiral) Alcohols

**Scheme 7:**
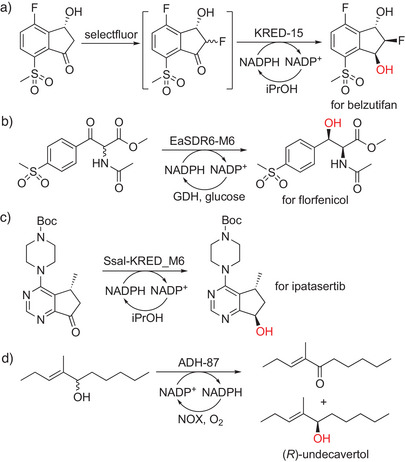
KRED‐catalyzed synthesis of chiral alcohols. a–c) Utilization of (engineered) KREDs to produce key pharmaceutical intermediates, and d) the value‐added flavor compound (*R*)‐undecavertol.

Chiral alcohols are well accessed by asymmetric reduction of the corresponding ketones by KREDs.^[^ ^108^, ^109^, ^110^, ^111^ ^]^ The current trends include the integration of KREDs with other chemical reactions and advanced semi‐rational enzyme engineering to produce key intermediates for new APIs. Merck & Co., Inc. developed a one‐pot chemoenzymatic ketone fluorination/reduction dynamic kinetic resolution (DKR) using an engineered KRED for the third‐generation synthesis of belzutifan (Scheme 7a).^[^ ^112^ ^]^

The desired *syn*‐1,2‐fluoroalcohol intermediate was produced in 85% isolated yield and >97% purity on a kilogram pilot scale. Another efficient KRED‐based DKR process was reported recently for chiral *syn*‐aryl *β*‐hydroxy *α*‐amino esters.^[^ ^113^ ^]^ Using an engineered KRED with improved activity and diastereoselectivity, another precursor for florfenicol was synthesized with >99%ee and >99%de from 300 g l ^−1^ substrate (Scheme 7b).

A recent example showed applying several advanced semi‐rational engineering strategies, including machine learning, to obtain a 10‐site substituted KRED variant, which enabled the production of the key chiral alcohol intermediate of ipatasertib with ≥98% conversion and 99.7%de from 100 g l ^−1^ substrate (Scheme 7c).^[^ ^114^ ^]^ Givaudan also reported the enzymatic synthesis of the flavor compound (*R*)‐undecavertol (99%ee), having the typical violet leaf and green cucumber smell, via kinetic resolution using an (*S*)‐selective alcohol dehydrogenase with NAD(P)H‐oxidase‐catalyzed recycling of the cofactor. At a substrate concentration of >400 g l ^−1^, a STY of 14 g l ^−1^ *h was achieved in the pilot plant, producing 85 kg of the target flavor compound (Scheme 7d).^[^ ^115^ ^]^

**Scheme 8:**
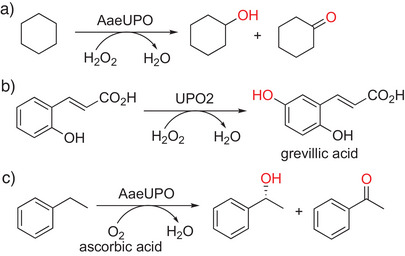
Peroxygenase‐catalyzed regio‐ and stereoselective hydroxylations. a) Up‐scaled production of cyclohexanol from cyclohexane by AaeUPO. b) The highly regiospecific hydroxylation of *o*‐coumaric acid by UPO2 yielded the value‐added grevillic acid. c) Synthesis of a chiral alcohol by AaeUPO, utilizing O_2_ and ascorbic acid (instead of H_2_O_2_).

Although cytochrome P450 monooxygenase (CYP)‐catalyzed reactions have been described to yield structurally diverse hydroxylated compounds, including alcohols but have yet often failed to become industrially viable, peroxygenases or α‐ketoglutarate‐dependent oxygenases represent interesting alternatives as pointed out by us in our 2021 review^[^ ^1^ ^]^ and by others.^[^ ^1^, ^2^, ^3^, ^116^, ^117^, ^118^ ^]^ Especially for unspecific peroxygenases (UPOs), high total turnover numbers (TTNs) have been reported. Recently, the first example showing that upscaling of a UPO‐catalyzed oxidation with H_2_O_2_ to the kilogram range is possible featured an enzyme from *Agrocybe aegerita* (AaeUPO), converting cyclohexane into cyclohexanol (58% yield, 29 g l ^−1^ product concentration, STY of 7.8 g l ^−1^ *h, TTN 33000), besides cyclohexanone as the second product (Scheme 8a).^[^ ^119^ ^]^ This reaction consumed 840 g lyophilized cell‐free extract (containing 33 g pure enzyme catalyst) per kg product. Unfortunately, the UPO inactivated quickly in this 11‐liter pilot experiment. The spatial separation of the H_2_O_2_‐dosing from the catalyst by use of an immobilized UPO in a rotating bed reactor, can be expected as a practical solution for increased UPO stability (TTN close to 10^6^ for ethylbenzene oxidation), as suggested by the Kara group.^[^ ^120^ ^]^ Another recent example for a highly regioselective hydroxylation reaction by an UPO is the synthesis of grevillic acid, a natural antioxidant, from *o*‐coumaric acid (Scheme 8b).^[^ ^121^ ^]^ A very recent study discovered an alternative catalytic route in haem peroxygenases, using O_2_ and a small‐molecule reductant such as ascorbic acid for specific oxidative transformations, thereby, avoiding the problematic addition of H_2_O_2_. Scale‐up reactions in 5‐L fermenter systems included the CYP‐catalyzed α‐hydroxylation of lauric acid (up to 12 g L^−1^ substrate load) and the UPO‐driven production of (*R*)‐1‐phenylethanol, starting from 200 mm ethylbenzene (Scheme 8c).^[^ ^122^ ^]^ Nonetheless, H_2_O_2_ remains a cheap and easy‐to‐dose oxidizing agent that can also be produced in situ by various strategies.^[^ ^123^, ^124^ ^]^

### 2.4 Enzymatic Alkylations for (Late‐Stage) Functionalization

**Scheme 9:**
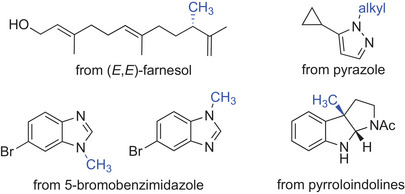
MT‐catalyzed *C*‐ or *N*‐alkylations. Different substrates were recently methylated or alkylated for the production of diverse bioactive molecules, including terpenoids (top left) and *N*‐heterocycles like pyrazoles (top right), benzimidazoles (bottom left), or a physostigmine derivative (bottom right).

In nature, alkylation reactions are essential steps in the biosynthesis of numerous metabolites and products, facilitated by glycosyltransferases, prenyltransferases, and methyltransferases (MTs).^[^ ^125^, ^126^ ^]^ Ospina et al. broadly summarized the fast progress made in the field of biocatalytic alkylation reactions.^[^ ^127^ ^]^ Among them, regioselective methylations play an important role in the manufacturing of natural products, including flavor compounds, as well as the synthesis of pharmaceuticals, especially for late‐stage functionalization. In this regard, *S*‐adenosyl‐methionine (SAM)‐dependent MTs have been heavily investigated since our review from 2021, which is also highlighted in two focused reviews, to overcome limitations in the practical use of MTs.^[^ ^128^, ^129^ ^]^ Drawbacks include the use of toxic methyl iodide, for example, which was addressed by the group of Seebeck, who suggested methyl tosylate^[^ ^130^ ^]^ as a safer alkylating agent. Further advancements catered microtiter‐plate based assays for the discovery and engineering of novel MTs,^[^ ^131^, ^132^ ^]^ as well as artificial regeneration systems for SAM.^[^ ^133^, ^134^, ^135^ ^]^ In this regard, the engineering of a halide methyltransferase (HMT)—previously used for the regeneration of SAM^[^ ^136^ ^]^—expanded the substrate scope of this enzyme beyond the acceptance of methyl iodide, as shown for alkylation reactions using ethyl, propyl, and allyl iodide to generate the corresponding SAM analogs. Combination with a suitable *O*‐MT achieved the late‐stage functionalization of various natural products.^[^ ^137^ ^]^ We could also demonstrate how engineered *O*‐MTs show different regioselectivity in the synthesis of the flavor compound hesperetin dihydrochalcone.^[^ ^138^ ^]^ The groups of Hammer and Hauer reported the engineering of a sterol MT for selective *C*‐methylation of linear terpenoids. The improved biocatalyst was able to methylate a range of non‐activated mono‐, sesqui‐, and diterpenoids with high chemo‐ and regioselectivity, yielding the corresponding C11, C16, and C21 derivatives (Scheme 9), most likely via a carbocation intermediate and subsequent regioselective deprotonation.^[^ ^139^ ^]^ In another example, the Hammer group performed the engineering of *N*‐MTs for *N*‐methylation and ethylation of heterocycles, including benzimidazoles, benzotriazoles, imidazoles, and indazoles. Using the HMT together with a suitable *N*‐MT and methyl tosylate, they demonstrated the highly regioselective synthesis of various products, including two 5‐bromobenzimidazole derivatives (Scheme 9).^[^ ^140^ ^]^ An example for selective *C*‐methylation was shown for the synthesis of physostigmine derivatives from pyrroloindolines recently.^[^ ^141^ ^]^ Finally, the *N*‐alkylation of pyrazoles was achieved by computational‐based enzyme design of a promiscuous MT. Pyrazole‐alkylating biocatalysts catalyzed methylation, ethylation, and propylation reactions with excellent regioselectivity and regiodivergence.^[^ ^142^ ^]^ Although admittedly far from larger‐scale applications, there certainly is interest by different industries, as pointed out already in our 2021 review.^[^ ^1^ ^]^

The examples discussed here demonstrate that only within a few years tools for the discovery of novel MTs acting on different heteroatoms have not only been developed: (engineered) biocatalysts were used—together with the efficient HMT‐based regeneration of SAM or the use of the less toxic tosylates as alkyl donors—to synthesize a broad variety of natural products and bioactive compounds.

### 2.5 (–)‐Ambrox^®^

**Scheme 10:**
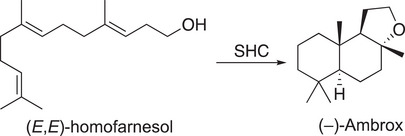
Synthesis of (–)‐Ambrox^®^ from (*E*,*E*)‐homofarnesol catalyzed by a squalene‐hopene‐cyclase (SHC).

The inherently high selectivities of enzymes can facilitate challenging transformations such as polyene cyclizations, in which multiple carbon–carbon bonds, ring systems, and stereogenic centers are formed in a single reaction step.^[^ ^143^, ^144^, ^145^ ^]^ One commercially relevant example is the synthesis of (–)‐Ambrox^®^ (synonyms: ambroxide, Ambroxan, Ambrofix; Scheme 10). It is the most prominent olfactive component of ambergris, has an ambery and woody odor, and is one of the most widely used biobased fragrance ingredients.^[^ ^146^, ^147^ ^]^ Already in 1986, Neumann and Simon found (–)‐Ambrox^®^ formation from *E,E*‐homofarnesol (EEH), catalyzed by a squalene–hopene–cyclase (SHC) from *Alicyclobacillus acidocaldarius*. Kao Corp.^[^ ^148^ ^]^ and BASF^[^ ^149^ ^]^ developed and patented improved processes based on EEH and SHC. The group of Hauer studied the working mode of SHC in depth and developed improved mutants.^[^ ^150^, ^151^ ^]^ The availability of the new feedstock (*E*)‐β‐farnesene produced by fermentation opened new routes to EEH as a precursor to (–)‐Ambrox^®^. Givaudan developed the chemical transformation of (*E*)‐β‐farnesene to EEH and the enzymatic cyclization using *E. coli* whole‐cells, overexpressing an engineered variant of SHC, to provide a new sustainable and scalable route for the production of (–)‐Ambrox^®^. With the third generation of SHC variants, 450 g l ^−1^ of the substrate EEH can be fully converted to the target molecule within 72 h, using 180 g l ^−1^ whole‐cell biocatalyst.^[^ ^152^ ^]^ Compared to the route from sclareol,^[^ ^153^ ^]^ a diterpene alcohol isolated from the herbal and flavor plant clary sage, the new process improved atom and step economy, reduced waste production, as well as solvent and energy consumption.

### 2.6 Glycosylation and Complex Carbohydrates

Once harnessed, the enzymatic transformation of challenging substrates like terpenoids, as highlighted by the new synthetic routes toward (–)‐Ambrox^®^ above, has great potential for the modification and manufacturing of complex molecules. In this regard, carbohydrates are of interest to different industries.

Carbohydrates are the largest and versatile class of natural compounds and are important building blocks for other biopolymers, including DNA and RNA, as discussed below. Sugars exhibit a broad variety of biological functions, including pathology and disease, and are target molecules of the pharmaceutical industry.^[^ ^1^, ^2^, ^154^, ^155^ ^]^ However, carbohydrate synthesis and transformation are particularly challenging for conventional organic synthesis due to the presence of multiple, chemically similar alcohol functionalities. Hence, a plethora of protecting group chemistry is required to dictate selective bond formation and to prevent undesired side‐reactions, rendering organic synthetic schemes atom‐inefficient, wasteful, and costly.^[^ ^156^, ^157^ ^]^ Opportunities to address these issues are the utilization of the four classes of carbohydrate‐synthesizing enzymes—glycosyltransferases (GTs), transglycosidases/ hydrolases (TGs), glycosylphosphorylases (GPs), and artificial glycosynthases (GSs). These biocatalysts have been highlighted in the past^[^ ^158^ ^]^ and comprehensively reviewed for the example of (poly‐)‐d‐glucans very recently.^[^ ^159^ ^]^ As shown with the following examples, GPs have become a particularly pivotal instrument for carbohydrate conversions.

**Scheme 11:**
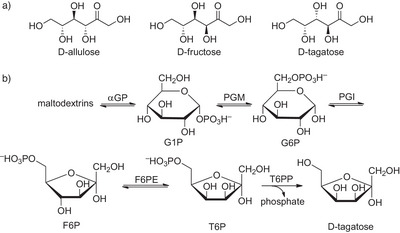
Industrial sugars. a) Structures of d‐allulose, d‐fructose, and d‐tagatose. b) d‐Tagatose production through an enzymatic cascade starting from maltodextrin. α‐GP: 1,4‐α‐glucan phosphorylase; PGM: phosphoglucomutase; PGI: phosphoglucoisomerase; F6PE: fructose‐6‐phosphate‐4‐epimerase; T6PP: tagatose‐6‐phosphate phosphatase; G1P: glucose‐1‐phosphate; G6P: glucose‐6‐phosphate; F6P: fructose‐6‐phosphate; T6P: tagatose‐6‐phosphate.

Although the production of fructose with immobilized glucose isomerase (EC 5.3.1.5) remains the largest biocatalytic process, the risk of metabolic diseases associated with high glucose, fructose, or sucrose consumption has spurred the interest for non‐ or low‐caloric substitutes that convey sugar‐like properties in beverages and foods (e.g., a clean sweet taste, familiar texture, and brown crusts in baking through the Maillard reaction).^[^ ^160^ ^]^ Besides high‐potency sweeteners (see below), a few “rare” sugars with low‐caloric profiles compared to table sugar (sucrose) are now being produced enzymatically on annual multi‐thousand tons scale: allulose, tagatose, lactulose, as well as cellobiose (see Scheme 11a).

Allulose, also known as psicose, can be obtained from fructose through immobilized d‐psicose‐3‐epimerase (EC 5.1.3.30),^[^ ^161^ ^]^ with free or immobilized resting cells overexpressing heterologous epimerases,^[^ ^162^, ^163^, ^164^ ^]^ or a free thermostable d‐tagatose 3‐epimerase (EC 5.1.3.31), which is retained in a membrane reactor.^[^ ^165^ ^]^ The different enzymes in use and advantages over production in metabolically engineered *E. coli* were discussed in recent reviews.^[^ ^166^, ^167^ ^]^ Usually, the epimerization leads to a 30:70 equilibrium allulose/fructose. To separate the two ketoses, a chromatographic separation is needed as elaborately investigated by the group of Panke.^[^ ^168^ ^]^ A subsequent crystallization process delivers pure allulose.^[^ ^169^ ^]^

Original processes toward d‐tagatose use l‐arabinose isomerase to isomerize d‐galactose (obtained from lactose) to d‐tagatose (CJ CheilJedang)^[^ ^170^ ^]^ or d‐fructose‐4‐epimerase to epimerize d‐fructose to d‐tagatose (see Scheme 11a).^[^ ^171^ ^]^ Complementary, Bonumose LCC designed a cascade process, using seven thermostable enzymes (immobilized on one column) to convert maltodextrins to d‐tagatose (Scheme 11b). Key enzymes are an 1,4‐α‐glucan phosphorylase (αGP) that provides glucose‐1‐phosphate (G1P), a phosphoglucomutase (PGM) that isomerizes G1P to glucose‐6‐phosphate (G6P), and a phosphogluco‐isomerase (PGI) to provide fructose‐6‐phosphate (F6P) from G6P. Subsequently, a fructose‐6‐phosphate‐4‐epimerase (F6PE) isomerizes F6P to tagatose‐6‐phosphate (T6P). Ultimately, T6P is selectively dephosphorylated by a selective tagatose‐6‐phosphate phosphatase (T6PP).^[^ ^172^ ^]^ The advantage of this cascade is the preservation of the energy content of the anomeric bonds in maltodextrin and the intermediate phosphates, only released in the last, T6PP‐catalyzed reaction step. Thus, the d‐tagatose content in the crude product is higher than in established isomerization processes. A chromatographic purification of the crude product is still needed, though.

Lactulose is a disaccharide produced for its prebiotic properties and as treatment for constipation. It can be formed by transgalactosidation of fructose or isomerization of lactose. The progress in optimization of cellobiose‐2‐epimerases for the production of lactulose (and epi‐lactose) from lactose has been reviewed very recently.^[^ ^173^ ^]^

**Scheme 12:**
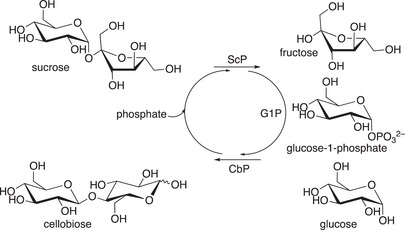
Reaction scheme for cellobiose synthesis. ScP, sucrose phosphorylase; CbP: cellobiose phosphorylase; G1P: glucose‐1‐phosphate.

Nidetzky et al. showed the power of full kinetic modelling to optimize the GP‐catalyzed synthesis of cellobiose under industrial conditions (Scheme 12)—uncovering inhibitory effects like microviscosity becoming relevant at high sugar concentrations (>100 g l ^−1^).^[^ ^174^ ^]^ Cellobiose is now produced and marketed by Savanna Ingredients for cosmetic formulations and as food texturizer with prebiotic benefits; cellobiose is also a potential pharma excipient as a replacement for lactose.^[^ ^175^ ^]^

Progress in the manufacturing of rebaudioside M, the most relevant high‐potency sweetener of the steviol glucosides, has been reviewed by Zhou and coworkers.^[^ ^176^ ^]^

### 2.7 Nucleoside and Nucleotide Analogs

Biocatalysis has certainly delivered molecules with increasing complexity and extended carbon–carbon frameworks through both the combination of enzymes in cascade‐type reactions and late‐stage functionalization.^[^ ^1^, ^2^, ^3^, ^117^ ^]^ Major progress has also been made in the manufacturing of nucleoside and nucleotide analogues with high relevance for pharmaceutical applications.^[^ ^1^, ^2^, ^3^, ^117^, ^177^ ^]^ A hallmark was the synthesis of islatravir, a deoxyadenosine analogue, and drug for the treatment of HIV.^[^ ^178^ ^]^ Initially produced from simple substrates by the combination of nine (engineered) enzymes, this multi‐enzyme cascade has been further optimized by Merck & Co., Inc. and Codexis,^[^ ^179^ ^]^ also giving valuable insights into the complex activation of galactose oxidase.^[^ ^180^ ^]^ The synthesis of the antiviral drug molnupiravir (MK‐4482) has seen different synthetic biocatalytic approaches.^[^ ^181^ ^]^ A three‐step synthesis, starting from ribose and uracil, employed kinases to phosphorylate ribose and recycle ATP and a uridine phosphorylase to build the nucleoside analog.^[^ ^182^ ^]^ Although this route yielded molnupiravir with 69% isolated yield, an alternative two‐step synthesis, utilizing the well‐available substrate cytidine and the lipase CAL‐B as biocatalyst, produced the target compound with 60% isolated yield.^[^ ^183^ ^]^

**Scheme 13:**
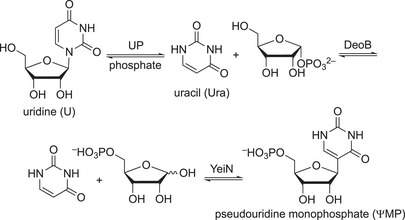
Manufacturing of ΨMP. A three‐enzyme cascade—combining an uridine phosphorylase (UP), a phosphopentamutase (DeoB), and a nucleoside *C*‐glycosylase (YeiN)—was developed to synthesize ΨMP.^[^ ^186^ ^]^

The need to efficiently produce and modify messenger RNA (mRNA) has gained a boost due to the outbreak of COVID‐19 and the development of mRNA‐based vaccines.^[^ ^184^ ^]^ mRNA is considered an ideal non‐viral gene replacement tool with inherent advantages, including efficient transduction of primary cells and rapid protein production at low immunogenicity in vivo.^[^ ^185^ ^]^ Particularly, 5‐ribosyluracil, better known as pseudouridine (Ψ), has been shown to exhibit a stabilizing effect on (synthetic) mRNAs, thus, is of high commercial interest. In a spectacular cascade development, combining a uridine phosphorylase, a phosphopentamutase, and a nucleoside *C*‐glycosylase, Nidetzky and coworkers isomerized uridine to Ψ and its 5′‐monophosphate (ΨMP). Their set‐up yielded ΨMP at 1.0–1.7 m concentration (up to 550 g l ^−1^, up to 98% conversion, >95% yield, with an STY of 30 g l ^−1^ *h; Scheme 13).^[^ ^186^ ^]^

Through the implementation of a selective phosphatase, phosphate is needed only in catalytic quantity and the free Ψ could be crystallized directly from the cascade batch, which further improved the STY to 36 g l ^−1^ *h at only 8 g enzyme consumption per kg product due to the reduced product inhibition of DeoB. UAB Biomatter and EnginZyme AB have jointly filed a patent for this route for commercial exploitation.^[^ ^187^ ^]^ Noteworthily, the phosphorylation of ribose (or deoxyribose) by ribose kinase and subsequent reaction with free uracil can be an alternative entry to this cascade.^[^ ^188^ ^]^

The manufacturing of the cyclic dinucleotide ulevostinag (MK‐1454)^[^ ^189^ ^]^ is another example for advanced cascades featuring novel combinations of highly evolved enzymes for artificial nucleotides.^[^ ^190^ ^]^ These developments facilitate the enzymatic synthesis and customization of DNA and particularly chemically modified RNA oligonucleotides as discussed below. The synthesis of both relies on the supply of (artificial) nucleoside triphosphates (NTPs) building blocks. Nucleosides functionalized at the 2′‐position are of special interest since they serve as both small‐molecule drugs and synthons for therapeutic oligonucleotides.^[^ ^191^ ^]^ Their synthesis has been realized recently through (engineered) enzymatic cascades, also yielding 2ʹ‐fluoro oligonucleotides.^[^ ^192^ ^]^ The access to 2′‐modified nucleotides, which can be found in approved therapeutic siRNA (e.g., lumasiran or inclisiran)^[^ ^191^, ^193^ ^]^ further demonstrates the diversity of substrates that can be accessed and modified by a continuously growing enzymatic toolbox,^[^ ^194^ ^]^ the use of biocatalytic cascades,^[^ ^195^ ^]^ and the promise of scalability^[^ ^196^ ^]^ for industrial applications.

### 2.8 DNA and RNA Syntheses

The demand for DNA and RNA availability on industrial scale is rapidly growing across many sectors of research and commercial technologies, with a desire to come up with a more sustainable production than the well‐established, solid‐phase synthesis based on phosphoramidite chemistry.^[^ ^197^ ^]^

Although DNA reading has advanced, driven by human genome sequencing campaigns in early 2000s, major progress and alternative synthetic technologies in DNA writing are highly demanded to close the reading–writing gap. Around the same time of completing the human genome sequencing, RNA interference (RNAi) was discovered for therapeutic applications, representing a breakthrough discovery for a new class of medicines.^[^ ^198^, ^199^ ^]^

The inherently mild reaction conditions of enzymatic approaches are perfectly suitable for the chemical manufacturing of (modified) nucleosides building blocks. A major advantage, in contrast to established chemical synthesis and as discussed above for other molecules, is the reduction—even omission—of protecting group chemistry owed to the high selectivity of biocatalysts, contributing to better atom economy balance as reviewed recently for oligonucleotide synthesis.^[^ ^200^ ^]^ However, the bar for implementation of an industrially relevant, alternative synthesis is very high due to excellent coupling efficiency of >99.5% achieved during solid‐phase synthesis.^[^ ^200^, ^201^ ^]^

A potential alternative could be the iterative process of a polymerase‐catalyzed extension of DNA by addition of 2′‐deoxy‐NTPs (dNTPs).^[^ ^202^ ^]^ To control the run‐away polymerization, the hydroxyl group at the 3′‐position can be reversibly blocked by a suitable protecting group. In the following hydrolytic step, the 3′‐position is liberated and ready for the next cycle of dNTP addition. For the manufacturing purposes of DNA, those enzymes need to be improved by enzyme engineering in order to remain competitive with chemical synthesis. Native terminal deoxynucleotidyl transferases (TdTs) react slowly with blocked dNTPs. Hence, new TdTs were discovered and engineered for this new application.^[^ ^203^ ^]^ These new TdT‐based approaches have driven DNA oligonucleotide synthesis to (literally) new lengths of up to 1000 nucleotides. Incorporation efficiencies of >99.6% have renewed the promise for a rapid, single‐run whole‐gene synthesis through biocatalysis. Codexis recently reported on the evolution of a TdT with significant improvements to stability, solubility, and expression achieved within 32 rounds of evolution. The accumulation of 80 mutations yielded a TdT with increased thermostability (+24 °C) and an impressively reduced reaction time from 16 h to 90 s.^[^ ^204^ ^]^ Such a concept using various blocking groups has been used also for template‐independent synthesis of RNA.^[^ ^205^ ^]^ To control the run‐away polymerization, Ansa Biotechnologies came up with technology using modified nucleobases instead of 3′‐*O*‐reversible terminators. In this case, the TdT enzymes are directly tethered to the nucleobase of a nucleotide through a linker arm. Directly connecting the TdT to the nucleobase permits the incorporation of a single, specific nucleotide into DNA and concomitantly accelerates the incorporation rate.^[^ ^202^ ^]^ Using this technology, Ansa Biotechnologies announced successful de novo synthesis of an oligonucleotide with >1000 bases.

An alternative concept to the methods above is the template‐dependent synthesis of antisense oligonucleotides, including fomivirsen and its conjugates, which are used as antiviral drugs.^[^ ^206^ ^]^ In this approach, DNA is used as template and shorter fragments are ligated together by a T3 DNA ligase, as demonstrated in a collaboration of the group of Hollenstein with researchers at Roche.^[^ ^207^ ^]^ An orthogonal approach toward clinically relevant therapeutics was demonstrated by the team of Lovelock, who used a smart design of catalytic self‐priming templates, in combination with a polymerase and an endonuclease in one pot.^[^ ^208^ ^]^

A successful demonstration of different chemoenzymatic routes toward both sense and antisense strands of inclisiran was presented by Codexis as proof‐of‐concept of their enzymatic toolbox developed for the manufacturing of RNA.^[^ ^209^ ^]^ Inclisiran is used for the treatment of patients with high levels of low‐density lipoprotein (LDL) cholesterol, which can be related to atherosclerotic cardiovascular diseases and types of hypercholesterolemia.^[^ ^210^ ^]^ The drug is a small interfering RNA (siRNA), conjugated to *N*‐acetyl galactosamine (GalNAc) that inhibits the translation of the protein PCSK9, which impedes the uptake of LDL from the blood.^[^ ^211^, ^212^ ^]^ In addition to the synthesis, Codexis also demonstrated the enzymatic ligation of siRNA and GalNAc. Almost quantitative conversion and very good purity of duplex products were achieved, setting a solid foundation for further improvements in volumetric productivity and potential transfer to a larger‐scale production.

Currently, several companies are developing own approaches toward oligonucleotide synthesis. This is driven by recent advancements in oligonucleotide drug delivery^[^ ^213^ ^]^ and the potential for a sequence‐specific, precise, and personalized treatment of not only rare diseases but widespread, chronic conditions and applications in combination therapies.^[^ ^214^, ^215^ ^]^ However, truly large‐scale production of siRNAs and related therapeutics is yet to be established in a hopefully not so distant future. The excellent overview regarding the (chemo‐)enzymatic synthesis of oligonucleotides by Bizat et al.^[^ ^200^ ^]^ may serve as a template to guide the next developments in oligonucleotide production that should also target the enzymatic conjugation of oligonucleotides to its respective targeting moiety. To date, the majority of oligonucleotide therapeutics, as well as approved nucleic acid drugs, have focused on either local delivery (e.g., to the eye or the spinal cord) or to the liver.^[^ ^213^ ^]^ Chemical modifications represent one of the most effective approaches to address this issue and feature backbone alterations (e.g., incorporation of phosphorothioate linkages^[^ ^216^ ^]^), nucleobase decorations (e.g., methylation), and modifications at the 2ʹ‐position of ribose sugar moieties, besides others. Many of these transformations can be facilitated by (engineered) biocatalysts and have been highlighted above. Furthermore, several alternative conjugates of antibodies or peptides and siRNAs have already passed clinical trials.^[^ ^199^ ^]^ Precise control over chemo‐, stereo‐, and regioselectivity is key in the synthesis of DNA and RNA molecules—three excellent reasons why enzymatic catalysis might become a prominent method of choice in this emerging market.

### 2.9 Enzymatic Plastic Depolymerization

Although we highlighted enzyme‐catalyzed (cascade‐type) reactions to manufacture complex molecules above, this section will aim at grasping future trends in enzyme‐based bond‐breaking reactions with potential for industrial applications such as the biocatalytic degradation of (synthetic) polymers.

The latter is of interest in the context of plastic pollution. Since the 1950s, more than ten billion tons of plastics have been manufactured and the current production scale is >450 million tons per year. Consequently, the accumulation of plastic waste is of major environmental concern worldwide^[^ ^217^, ^218^ ^]^ and considerable efforts have been made to degrade and recycle commodity plastics through biocatalytic approaches.^[^ ^219^ ^]^ Plastics that still largely resist biodegradation (e.g., by microbes) include poorly functionalized polymers with carbon–carbon backbones, like polypropylene (PP) or polyethylene (PE).^[^ ^220^ ^]^ Although enzyme cascade‐based conversions for a polyvinyl alcohol^[^ ^221^ ^]^ and a low‐molecular weight PE^[^ ^222^ ^]^ have been recently reported, the need for chemical functionalization prior to enzymatic treatment, the low price of virgin polymers, and the impossibility to access defined building blocks for closed‐ and open‐loop recycling make it unlikely that economical bio‐based routes for polyolefins will be developed soon. Hence, energetic use or production of syngas might be the most straightforward ways to use these types of plastic waste.

In contrast, synthetic polymers like polyesters, polyamides (PAs), and polyurethanes (PUs) have hydrolyzable backbones and their enzyme‐based depolymerization has greatly advanced since the discovery of (poly)ester hydrolases, for example, in metagenomic samples.^[^ ^219^, ^223^, ^224^ ^]^ Their customization for process conditions was achieved through structure‐based and bioinformatic‐guided protein engineering approaches.^[^ ^225^, ^226^ ^]^ Improved (poly)ester hydrolases significantly accelerate the depolymerization of polyethylene terephthalate (PET), one of the most commonly used synthetic ester‐based polymers.^[^ ^225^, ^226^ ^]^ Currently, the process by the company Carbios is the most advanced and utilizes an engineered variant of the leaf and branch compost cutinase (LCC‐ICCG).^[^ ^225^ ^]^ The Carbios process has been evaluated in a thorough techno‐economic life‐cycle‐analysis (LCA), which came to an overall positive evaluation; however, under the assumption of low enzyme cost (15 € per kg biocatalyst) and selling the by‐product sodium sulfate (almost one ton per ton recycled TPA).^[^ ^227^ ^]^ An industrial plant, allowing the bio‐based recycling of 50,000 tons of post‐consumer PET per year is scheduled to start in 2026.^[^ ^228^ ^]^ Critical process assessments and LCA are particularly important regarding the biotechnological depolymerization, re‐ and upcycling of PET (and other plastics), as suggested and reviewed by various groups.^[^ ^229^, ^230^, ^231^, ^232^, ^233^, ^234^ ^]^ These processes have to compete with alternative strategies like methanolysis—which only needs small (i.e., catalytic) amounts of base^[^ ^235^, ^236^ ^]^—besides other chemical, mechanical, or hybrid recycling schemes.^[^ ^234^, ^237^, ^238^, ^239^ ^]^ Nonetheless, (bio)chemical depolymerization yields monomeric building blocks—terephthalic acid (TPA) and ethylene glycol (EG) in the case of PET—to manufacture virgin polymers (closed‐loop recycling) or other value‐added products (open‐loop recycling).^[^ ^230^, ^232^, ^240^ ^]^

In the context of waste valorization and open‐loop recycling, the enzyme‐based degradation of synthetic polymers other than PET is an emerging technology.^[^ ^219^, ^240^, ^241^, ^242^, ^243^ ^]^ Carbios developed a concept, where a polylactide (PLA) depolymerase is incorporated into a PLA film (0.02% (w/w) enzyme) that fully disintegrates under home‐compost conditions within about 20 weeks. To achieve this, extensive protein engineering of the PLA hydrolase was performed, resulting in an 80‐fold activity enhancement. The improved biocatalyst was blended into polycaprolactone (PCL) at 70 °C. The resulting PCL pellets were manufactured into PLA by a melt extrusion process carried out at 160 °C. Impressively, the entrapped hydrolase retained activity, enabling the biodegradation of PLA films during composting.^[^ ^244^ ^]^

**Scheme 14:**
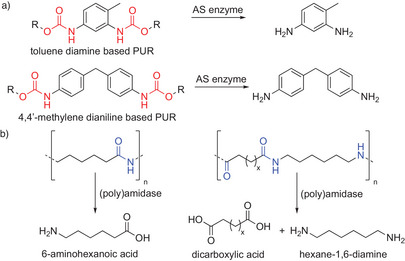
Common building blocks accessible from the depolymerization of PUs and PAs (nylons). a) Hydrolysis of toluene diamine‐based PUs (top) and 4,4′methylene dianiline‐based PUs (bottom) by AS family members yields the corresponding monomers 2,4‐toluene diamine and 4,4′‐methylene dianiline, respectively. b) NylC‐type nylonases and AS enzymes are currently investigated for their (poly)amidase activity toward different PAs. Hydrolysis of nylon 6 produces 6‐aminohexanoic acid (left). The depolymerization of nylon 4,6 (x  =  1) and nylon 6,6 (x  =  3) yields hexane‐1,6‐diamine, besides succinic acid and adipic acid, respectively (right).

Other polymers with hydrolyzable backbones are PAs and PUs.^[^ ^241^, ^245^, ^246^ ^]^ Although PA‐ and PU‐degrading organisms, as well as various (poly)esterases and amidases, have been reported in the past, only basal activity could be detected on PA‐oligomers and (mixed) polyesters/(poly)urethanes; selected building blocks are shown in Scheme 14.^[^ ^242^, ^247^, ^248^, ^249^, ^250^, ^251^, ^252^ ^]^ This shortcoming could be addressed after the discovery of three metagenome‐derived urethanases UMG‐SP‐1, UMG‐SP‐2, and UMG‐SP‐3.^[^ ^253^ ^]^ That UMG‐SP‐1–3 can act on different PAs and PUs—as the sole biocatalysts or in tandem with other enzymes—was demonstrated only recently by quantifying the release of the corresponding monomeric building blocks (Scheme 14).^[^ ^241^, ^245^, ^254^ ^]^ Besides metagenomic urethanases, various (engineered) NylC‐type “nylonases”, which belong to the N‐terminal nucleophile hydrolase superfamily, are currently investigated for the depolymerization of different nylon polymers.^[^ ^241^, ^255^ ^]^

The heterologous expression of nylonases enabled *P. putida* to metabolize (linear and cyclic) nylon oligomers that had been derived from the chemical hydrolysis of PAs. To demonstrate microbial upcycling, polyhydroxybutyrate (PHB) was produced from nylon 6 hydrolysates (open‐loop recycling).^[^ ^256^ ^]^

Based on sequence homology, all metagenomic urethanases belong to the AS superfamily, briefly introduced above.^[^ ^257^, ^258^, ^259^ ^]^ AS family members exhibit a wide range of substrate specificities and biochemical functions. Although most act as amidases, some catalyze amide transfer reactions in complex with other proteins, or can act as acyltransferases as discussed above, and show protease, esterase, or the newly assessed urethanase activity.^[^ ^245^, ^253^, ^260^, ^261^, ^262^, ^263^ ^]^ This versatility and their capability to hydrolyze highly stable *N*‐aryl amide and carbamate bonds present in small molecules and polymeric materials (Scheme 14), make this class of amidases an interesting target for extensive protein and process engineering to advance future industrial applications.

### 2.10 Enzymatic Deprotection

One strategy—albeit not yet realized for AS family members but demonstrated for related α/β‐hydrolases—is the enzyme‐catalyzed removal of chemical protecting groups. This has long been of interest^[^ ^264^ ^]^ and was revisited by the group of Campopiano recently. They reported the successful use of an esterase from *Bacillus subtilis* ^[^ ^265^ ^]^ to remove a *tert*‐butyl group, protecting the carboxyl group of amino acids. Interestingly, an amidohydrolase cleaved a benzyloxycarbonyl (Cbz or Z) group, masking the amino functionality in the same molecule in aqueous solution under mild reaction conditions.^[^ ^266^ ^]^ Even though the protection and deprotection of functional groups—not limited to amines^[^ ^267^ ^]^—introduces uneconomical steps into synthetic schemes, protecting groups are irreplaceable in multi‐step organic synthesis to prevent undesired off‐target reactions.^[^ ^268^, ^269^, ^270^ ^]^ However, if a protecting group strategy is well thought through, the utilization of a regioselective amidohydrolase may offer the possibility for desymmetrization if two Cbz‐protected amines are present in the same molecule. Similarly, the chemoselectivity of Cbz‐cleaving enzymes ensures removal of the Cbz‐group while leaving, for example, a Boc‐group untouched. With various biocatalysts available, transforming amines into the corresponding amides^[^ ^60^, ^73^, ^74^, ^75^ ^]^ or carbamates,^[^ ^271^ ^]^ we envision the realization of fully enzymatic protection/deprotection schemes in the near future, especially for the synthesis of complex natural products and pharmaceuticals, including (therapeutic) peptides.^[^ ^267^, ^272^, ^273^, ^274^ ^]^ Similarly, the combination of naturally unrelated MTs and oxidoreductases such as CYPs^[^ ^275^, ^276^, ^277^ ^]^ can be used for the protection and deprotection, respectively, of hydroxyl and amino functionalities, for example.

## 3 Conclusion and Outlook

The presented update highlights a considerable number of novel biocatalytic applications that have matured into industrially viable and commercialized processes within only a few years as shown for the RedAm‐catalyzed synthesis of the LSD1 inhibitor GSK2879552.^[^ ^278^ ^]^ Although we might have missed some very recent examples and could not elaborate on important biosynthetic routes, yielding the natural antibiotic ikarugamycin^[^ ^279^ ^]^ or novel insulins,^[^ ^280^ ^]^ we were impressed by the number of novel enzyme classes and engineered biocatalysts and their combination in cascade‐type reactions to perform currently challenging transformations, including DNA/RNA/oligonucleotide synthesis or the depolymerization of PET. Although not discussed herein in detail, the advancement of computational tools (recognized with the Nobel Prize in 2024) has accelerated the discovery of novel enzymes and facilitated the prediction of beneficial mutations in enzyme engineering campaigns. Besides these improved methods, bioprocess engineering will be crucial to implement more enzyme‐based processes in industry. Consequently, the vast progress in this research area has been summarized in several reviews. These highlight ongoing efforts in bioprocess intensification, featuring upstream operations, bioreactor/fermentation designs, and downstream separation steps, which—together with the aforementioned enzyme engineering—resulted in significant productivity enhancements.^[^ ^281^ ^]^ Remaining challenges include the mass transfer at liquid‐liquid and gas–liquid interfaces, but also known biocatalyst properties like stability and kinetic parameters to be addressed under industrially relevant conditions^[^ ^282^, ^283^ ^]^ to meet key metrics—titer, rate, yield, and space–time‐yield.^[^ ^1^, ^284^ ^]^ These numbers are important to assess the economic feasibility and were summarized for various biocatalytic reactions operating at industrial scales.^[^ ^1^, ^285^, ^286^ ^]^ When key players from several companies in the fine chemicals, fragrance and flavor industries were asked about the potential of biocatalysis, a unified response emphasized the potential of biocatalytic cascades, combinations of chemo‐ and biocatalytic multi‐step transformations, and novel biocatalytic transformations, to significantly shorten synthetic routes and improve synthetic efficiency, given that biocatalysts are available in commercial quantities and easy to implement.^[^ ^287^, ^288^ ^]^ The various examples in this update certainly support this trend. Although most of the shown examples were developed for interconversion of functional groups, more research and development are still needed in making new bonds and in direct functionalization of complex molecules. With recent renewed interest in peptides (for targeting and therapeutic purposes) and for DNA/RNA, we will need to continue on our journey toward expansion of an even larger enzymatic toolbox to tackle future challenges, which the era of complex molecules is imposing on synthetic chemists.

Together with the rapid technological advancements, we are confident that biocatalysis is very well on its way to reliably, sustainably, and cost‐competitively delivering value‐added and highly demanded molecules on industrial scales, thereby tackling today's and future medical, socioeconomic, and environmental challenges.

## Conflict of Interests

The authors declare no conflict of interest.

## Data Availability

Data sharing is not applicable to this article as no new data were created or analyzed in this study.
